# Powerful expression in Chinese Hamster Ovary cells using bacterial artificial chromosomes: parameters influencing productivity

**DOI:** 10.1186/1753-6561-7-S6-P25

**Published:** 2013-12-04

**Authors:** Wolfgang Sommeregger, Andreas Gili, Thomas Sterovsky, Emilio Casanova, Renate Kunert

**Affiliations:** 1 Vienna Institute of BioTechnology (VIBT), Department of Biotechnology, University of Natural Resources and Life Sciences, Vienna, 1190, Austria; 2 Polymun Scientific Immunbiologische Forschung GmbH, Klosterneuburg, 3400, Austria; 3 Ludwig Boltzmann Institute for Cancer Research (LBI-CR), Vienna, 1090, Austria

## Background

CHO (Chinese Hamster Ovary) cells are the cell line of choice for therapeutic protein production. Although the achieved volumetric titers have increased significantly over the past two decades, the establishment of well-producing CHO cell lines is still difficult and not always successful [1]. Factors influencing productivity are the chosen host cell line, the genetic vectors, applied media, the cultivation strategy as well as the product itself. Several CHO host strains are available for recombinant protein production, however, they are often quite diverse in terms of growth rate, maximal achieved cell concentrations and specific productivities. Specific productivity is also related to the locus of integration of the transgenes due to positional effects caused by the chromatin environment. Previously it was described that Bacterial Artificial Chromosomes (BACs) carrying the Rosa26 locus are advantageous for the recombinant protein production in CHO cells, enhancing the specific productivity compared to plasmid derived recombinant CHO cells [2-4]. In this project we aim to identify factors influencing volumetric productivity using different CHO hosts, Rosa 26 BACs as genetic constructs and suitable cell culture media. First, different commonly used CHO host cell lines were analyzed in various cell culture media to identify which host strain performs best. Secondly, we generated a recombinant cell line, producing the highly glycosylated HIV envelope protein gp140 as an example for a difficult to express model protein. Gp140 expression was compared to an already existing gp140 cell line generated by a plasmid vector as expression system.

## Methods

Cell culture: CHO-DUKX-B11 (ATCC-CRL-9096) and CHO-DG44 (life technologies) were serum-free cultivated in spinner flasks. CHO-K1 (ATCC-CCL-61) and CHO-S (life technologies) were serum-free cultivated in in shaker flasks.

BAC Recombineering: *E.coli *carrying the Rosa 26 BAC (~220 kbp) were transformed with a plasmid coding for a recombinase. Consecutively, a plasmid carrying the gp140 (CN54) gene flanked by homologous regions to the BAC was used for the transformation of the recombinase positive *E.coli *cells. BAC positive colonies were selected and the BAC DNA was purified (NucleoBond Xtra BAC, Macherey Nagel).

Transfection and selection: CHO-S host cells were transfected with linearized, lipid complexed (Lipofectin) CN54 Rosa26 BAC DNA. Recombinant clone selection was performed in 96-well plates using 0.5 mg/mL G418. BAC transfected CHO cells are able to express the transgene as well as a Neomycin resistance gene within the Rosa26 locus.

## Results

### Host cell line comparison

CHO-DUKX-B11, CHO-DG44, CHO-K1 and CHO-S were analyzed in batch culture in CD-CHO (life technologies), ActiCHO (GE-PAA), DMEM/Ham's F12 (Biochrom) + supplements (Polymun Scientific), and CD-DG44 (life technologies) media in spinner and shaker flasks. CHO-DUKX-B11 and CHO-DG44 grew best in spinner flasks with CD-DG44 media, whereas CHO-K1 and CHO-S grew best in shaker flasks with ActiCHO media. The dhfr negative cell lines were growing to much lower viable cell densities than K1 and S. CHO-S reached the highest viable cell density (1.17 × 10^7 ^cells/mL) followed by CHO-K1 (8.39 × 10^6 ^cells/mL) (Table 1).

**Table 1:** Maximum achieved viable cell densities in batch experiments.

CHO cell line	*DUKX-B11*	*DG44*	*CHO-S*	*CHO-K1*
Max. VCD (cells/mL)	2.00E+06	2.28E+06	1.17E+07	8.39E+06

### Gp140 (CN54) recombinant cell lines

**Figure 1:**
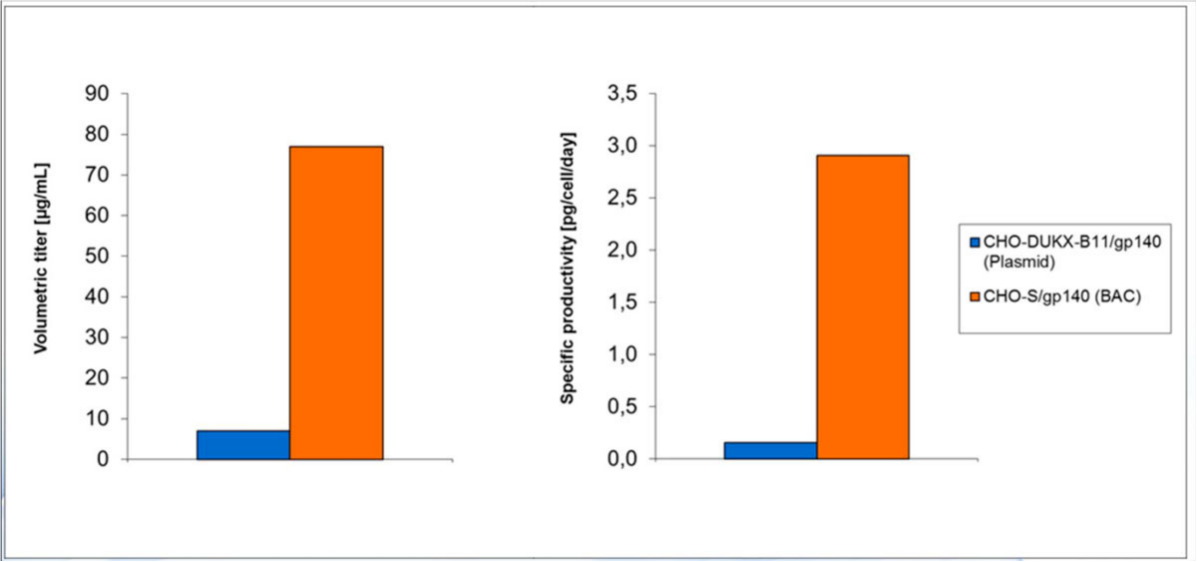
**Titer and specific productivity comparison of a BAC derived recombinant CHO-S cell line producing gp140 (CN54) and an already existing recombinant plasmid derived CHO-DUKX-B11 cell line**.

CHO-S was chosen for test-transfections and recombinant gp140 (CN54) producers were established using a Rosa 26 BAC construct carrying the gp140 (CN54) gene. The best clone was analyzed in a batch experiment and yielded 77 μg/mL which is ~10 times the titer achieved with a recombinant plasmid derived CHO-DUKX-B11 (Figure 1). This 10-fold increase was related to the higher specific productivity (~18-fold) and the higher accumulated cell density (3.5-fold) in shorter batch duration.

## Conclusion

CHO-S and CHO-K1 have the potential to grow to high cell densities. The used dhfr deficient hosts (DUKX-B11 and DG44) are at least without a co-transfection of the dhfr gene not growing to high cell concentrations. Rosa 26 BAC derived clones need no amplification as they provide their own open chromatin region. Thus, higher specific productivity can be achieved by elevated transcript levels compared to conventional plasmid clones. The combination of cells growing to high cell densities and the transcriptional efficiency of the Rosa26 BAC system leads to accumulation of significantly increased volumetric titers for a difficult to express glyco-protein.
